# Maternal Characteristics and Rates of Unexpected Complications in Term Newborns by Hospital

**DOI:** 10.1001/jamanetworkopen.2024.11699

**Published:** 2024-05-20

**Authors:** Kimberly B. Glazer, Jennifer Zeitlin, Natalie Boychuk, Natalia N. Egorova, Paul L. Hebert, Teresa Janevic, Elizabeth A. Howell

**Affiliations:** 1 Department of Population Health Science and Policy, Icahn School of Medicine at Mount Sinai, New York, New York; 2 Raquel and Jaime Gilinski Department of Obstetrics, Gynecology and Reproductive Science, Icahn School of Medicine at Mount Sinai, New York, New York; 3 Blavatnik Family Women’s Health Research Institute, Icahn School of Medicine at Mount Sinai, New York, New York; 4 Université Paris Cité, Inserm, Centre for Research in Epidemiology and Statistics, Obstetrical Perinatal and Pediatric Epidemiology Research Team, Paris, France; 5 School of Public Health, University of Washington, Seattle; 6 Department of Epidemiology, Columbia University Mailman School of Public Health, New York, New York; 7 Department of Obstetrics & Gynecology, Perelman School of Medicine, University of Pennsylvania, Philadelphia

## Abstract

**Importance:**

The Joint Commission Unexpected Complications in Term Newborns measure characterizes newborn morbidity potentially associated with quality of labor and delivery care. Infant exclusions isolate relatively low-risk births, but unexpected newborn complications (UNCs) are not adjusted for maternal factors that may be associated with outcomes independently of hospital quality.

**Objective:**

To investigate the association between maternal characteristics and hospital UNC rates.

**Design, Setting, and Participants:**

This cohort study was conducted using linked 2016 to 2018 New York City birth and hospital discharge datasets among 254 259 neonates at low risk (singleton, ≥37 weeks, birthweight ≥2500 g, and without preexisting fetal conditions) at 39 hospitals. Logistic regression was used to calculate unadjusted hospital-specific UNC rates and replicated analyses adjusting for maternal covariates. Hospitals were categorized into UNC quintiles; changes in quintile ranking with maternal adjustment were examined. Data analyses were performed from December 2022 to July 2023.

**Main Outcomes and Measures:**

UNCs were classified according to Joint Commission *International Statistical Classification of Diseases and Related Health Problems, Tenth Revision *(*ICD-10*) criteria. Maternal preadmission comorbidities, obstetric factors, social characteristics, and hospital characteristics were ascertained.

**Results:**

Among 254 259 singleton births at 37 weeks or later who were at low risk (125 245 female [49.3%] and 129 014 male [50.7%]; 71 768 births [28.2%] to Hispanic, 47 226 births [18.7%] to non-Hispanic Asian, 42 682 births [16.8%] to non-Hispanic Black, and 89 845 births [35.3%] to non-Hispanic White mothers and 2738 births [1.0%] to mothers with another race or ethnicity), 148 393 births (58.4%) were covered by Medicaid and 101 633 births (40.0%) were covered by commercial insurance. The 2016 to 2018 cumulative UNC incidence in New York City hospitals was 37.1 UNCs per 1000 births. Infants of mothers with preadmission risk factors had increased UNC risk; for example, among mothers with vs without preeclampsia, there were 104.4 and 35.8 UNCs per 1000 births, respectively. Among hospitals, unadjusted UNC rates ranged from 15.6 to 215.5 UNCs per 1000 births and adjusted UNC rates ranged from 15.6 to 194.0 UNCs per 1000 births (median [IQR] change from adjustment, 1.4 [−4.7 to 1.0] UNCs/1000 births). The median (IQR) change per 1000 births for adjusted vs unadjusted rates showed that hospitals with low (<601 deliveries/year; −2.8 [−7.0 to −1.6] UNCs) to medium (601 to <954 deliveries/year; −3.9 [−7.1 to −1.9] UNCs) delivery volume, public ownership (−3.6 [−6.2 to −2.3] UNCs), or high proportions of Medicaid-insured (eg, ≥90.72%; −3.7 [−5.3 to −1.9] UNCs), Black (eg, ≥32.83%; −5.3 [−9.1 to −2.2] UNCs), or Hispanic (eg, ≥6.25%; −3.7 [−5.3 to −1.9] UNCs) patients had significantly decreased UNC rates after adjustment, while rates increased or did not change in hospitals with the highest delivery volume, private ownership, or births to predominantly White or privately insured individuals. Among all 39 hospitals, 7 hospitals (17.9%) shifted 1 quintile comparing risk-adjusted with unadjusted quintile rankings.

**Conclusions and Relevance:**

In this study, adjustment for maternal case mix was associated with small overall changes in hospital UNC rates. These changes were associated with performance assessment for some hospitals, and these results suggest that profiling on this measure should consider the implications of small changes in rates for hospitals with higher-risk obstetric populations.

## Key Points

**Question:**

Do hospital rates of unexpected newborn complications (UNCs) change after adjustment for maternal characteristics?

**Findings:**

In this cohort study of 254 259 neonates at low risk, maternal risk adjustment was associated with modest changes in overall hospital UNC rates, with significant improvement after adjustment among hospitals with higher proportions of births to Black, Hispanic, or Medicaid-insured individuals. Changes were observed in the relative performance of hospitals ranked by unadjusted and adjusted UNC quintile.

**Meaning:**

These findings suggest that failure to adjust for maternal characteristics may be associated with less fair comparison for profiling on the UNC measure because even small changes in rates may be associated with differences in performance assessment and inappropriate penalties for hospitals with higher-risk obstetric populations.

## Teaser

This cohort study investigates whether adjusting for maternal characteristics is associated with changes in rates of unexpected newborn complications by hospital.

## Introduction

The Joint Commission adopted Perinatal Care Core Measure PC-06, Unexpected Complications in Term Newborns, in 2019 to measure adverse infant outcomes that may be associated with quality of labor and delivery care. Developed and validated by the California Maternal Quality Care Collaborative and endorsed by the National Quality Forum, this metric captures newborn morbidity in otherwise-healthy births. PC-06 is the first perinatal care measure for standardized tracking of newborn complications among births at 37 weeks or later, which constitute more than 90% of all deliveries, and serves as a balancing measure for other maternal-focused measures, such as the rate of low-risk (ie, nulliparous, ≥37 weeks, singleton, and vertex presentation) cesarean deliveries.^1^

Given the inclusion of PC-06 in the Perinatal Care measure set, hospitals are required to report performance on PC-06 for Joint Commission Perinatal Care certification. PC-06 isolates a subset of low-risk births by excluding serious fetal conditions, such as prematurity, congenital anomalies, and inadequate growth, meaning births not expected to have optimal outcomes. Because this measure was developed for use with infant records, measure criteria do not exclude or adjust for maternal factors that may be associated with birth outcomes even if they do not result in preterm delivery or preexisting fetal risks. Profiling hospitals on this measure therefore may not render fair comparisons among hospitals serving obstetric populations with different rates of medically complex pregnancies (eg, those with maternal diabetes or hypertension) or exposure to social or structural health risks (eg, environmental or neighborhood deprivation) unrelated to delivery care.^2,3,4^

Accordingly, our objective was to investigate the association of maternal characteristics with hospital rates of unexpected newborn complications (UNCs) in New York City (NYC). We hypothesized that hospital UNC performance would differ when using maternal risk–adjusted estimates as opposed to observed rates calculated from current PC-06 measure criteria.

## Methods

### Data Source

We conducted a retrospective, population-based cohort study of live births in NYC hospitals. The Statewide Planning and Research Cooperative System (SPARCS) provided discharge data on 330 945 births from 2016 to 2018. We linked SPARCS data from the maternal discharge record to birth certificate data using a deidentified person identifier (ID), excluding deliveries with missing ID, resulting in 326 632 observations. We further linked these records to discharge data for 309 507 singleton infants (eFigure 1 in Supplement 1). Institutional review boards (IRBs) of the NYC Department of Health and Mental Hygiene, New York State Department of Health, and Icahn School of Medicine at Mount Sinai approved the study protocol. The Icahn School of Medicine at Mount Sinai IRB granted a waiver of informed consent for use of this deidentified database. We followed the Strengthening the Reporting of Observational Studies in Epidemiology (STROBE) reporting guideline.

### Cohort Selection

From our sample of singleton deliveries, we excluded preterm (<37 completed weeks’ gestation) and low-birthweight (<2500 g) neonates and infants with congenital anomalies, genetic disorders, exposure to maternal drug use, or preexisting fetal or placental conditions, consistent with Joint Commission PC-06 restrictions (eFigure 1 in Supplement 1). We classified gestational age and birthweight using birth data, and we classified plurality, congenital disorders, fetal-placental conditions, and maternal drug use from *International Statistical Classification of Diseases and Related Health Problems, Tenth Revision *(*ICD-10*) diagnosis and procedures codes on the discharge record.^5,6^ In total, our study sample included 254 259 births in 39 hospitals. The delivery hospital was our exposure of interest.

### Outcome Variable

We identified UNCs using the Joint Commission *ICD-10* coding algorithm (eTable 1 in Supplement 1).^5,6^ We classified complications by type (neonatal death ≤28 days after birth, shock and resuscitation, transfer to a higher-level facility, birth trauma or neurologic complications, respiratory complications, and infection) and severity (severe vs moderate). All cases of neonatal death, shock, and transfer were classified as severe complications; birth trauma and neurologic, infection, and respiratory complications were further divided into severe vs moderate conditions. The algorithm additionally includes length of stay of 5 days or more in the absence of jaundice, phototherapy, or social needs as a moderate complication to account for potential undercoding in discharge data. UNC categories were mutually exclusive and assigned hierarchically such that a case of severe UNC could not also include moderate complications.

### Covariates

We ascertained maternal preadmission characteristics using *ICD-10* codes, the birth certificate, or both. Comorbidities (ie, preeclampsia, gestational hypertension, chronic hypertension, gestational diabetes, preexisting diabetes, pulmonary hypertension, asthma, pulmonary disease, bleeding disease, kidney disease, autoimmune condition, substance use disorder, anemia, bariatric surgery, major mental health disorder, neuromuscular disorder, and thyrotoxicosis) were defined from Leonard et al (eTable 2 in Supplement 1).^7,8,9^ Body mass index (BMI; calculated as weight in kilograms divided by height in meters squared) was ascertained from self-reported prepregnancy weight and height on the birth certificate and classified as underweight (BMI <18.5), normal weight (BMI, 18.5 to <25), overweight (BMI, 25 to <30), class 1 to 2 obesity (BMI, 30 to <40), and class 3 obesity (BMI ≥40). From vital statistics, we also measured previous cesarean delivery, age (<20, 20-34, or ≥35 years), parity (nulliparous or multiparous), education (<high school, high school diploma or General Educational Development test, or ≥some college), nativity (US vs not US), payor (Medicaid, private, uninsured, or other), and late entry into prenatal care (initiation after second trimester). Race and ethnicity were ascertained from maternal self-report on the birth certificate. We classified participants as Hispanic or not Hispanic based on selection of Cuban, Mexican, Puerto Rican, or other Hispanic origin. Participants who identified as Hispanic were classified as Hispanic, and race data were not included in categorization for these individuals. Race was classified among participants who identified as non-Hispanic ethnicity as Asian (inclusive of Asian Indian, Chinese, Filipino, Japanese, Korean, Vietnamese, or other Asian), Black (Black or Afro-American), White or Caucasian, or other race (American Indian or Alaska Native, Guamanian or Chamorro, Native Hawaiian, Other Pacific Islander, Samoan, or other race). Race and ethnicity were assessed to ascertain the racial and ethnic distribution of hospital obstetric populations and to examine UNC incidence by these characteristics.

We examined characteristics of the 39 hospitals, including public vs private hospital ownership (obtained from the New York State Department of Health) and total annual delivery volume (SPARCS). We calculated the percentage of Medicaid-insured deliveries and racial-ethnic distribution of births in each hospital and classified hospitals by quartile for each metric.

### Statistical Analysis

We calculated the 2016 to 2018 cumulative incidence of total, severe, and moderate complications over the study period by patient and hospital characteristic. We estimated bivariate associations between maternal characteristics and UNC incidence using logistic regression models with robust standard errors clustered at the hospital level.

#### Hospital Complication Rates

We estimated unadjusted UNC rates by hospital. For risk-adjusted UNC rates, we used logistic regression with fixed effects for each hospital and maternal characteristics described previously to estimate the marginal effect by hospital. To avoid embedding bias by controlling for potential differential care by race and ethnicity, we did not include race and ethnicity as a covariate; instead, we included many social and clinical factors associated with perinatal disparities. We did not include hospital characteristics, such as delivery volume, in risk adjustment given that our research objective was to examine the association of maternal characteristics with hospital UNC rates. Furthermore, including hospital factors in regression models may inappropriately adjust away differences related to quality of care.^10^ In a sensitivity analysis, we followed the Centers for Medicare & Medicaid Services (CMS) modeling approach for hospital profiling, which calculates stabilized, indirectly risk-standardized, hospital-specific rates using hierarchical, mixed-effects logistic regression.^10^ These estimates account for patient clustering within hospitals and reduce random outcome variability, particularly in low-volume facilities. Unadjusted models included a hospital-specific random intercept only, and adjusted models included a hospital random intercept and controlled for the same set of maternal covariates as our primary models.

We examined the median (IQR) change in postadjustment vs preadjustment PC-06 rates for all hospitals. We also calculated unadjusted and adjusted median UNC rates within subgroups by hospital characteristic (eg, public vs private and delivery volume) and used the Wilcoxon rank sum test to compare differences within hospital subgroups before and after adjustment. Tests were 2-sided, with α = .05 used to denote statistical significance. Analyses were conducted in SAS statistical software version 9.4 (SAS Institute). Data were analyzed from December 2022 to July 2023.

#### Exploratory Hospital-Profiling Analyses

We carried out a set of analyses to investigate whether including maternal risk adjustment in PC-06 had implications for hospital profiling. We used 3 methods to compare relative hospital performance. First, we followed the methodology used by the *U.S. News & World Report* media company in its annual assessment of the Best Hospitals for Maternity Care. That scoring system uses quintile benchmarks, empirically derived from nationwide self-reported hospital data in the *U.S. News & World Report* Maternity Services Survey, to include UNC in a composite score of 8 maternity care indicators.^11^ We evaluated whether maternal risk adjustment was associated with UNC scoring by calculating quintiles of unadjusted and adjusted UNC rates among the 39 NYC hospitals, categorizing each hospital’s unadjusted rate by quintile, recategorizing the rate after adjustment, and investigating whether adjustment was associated with quintile assignment. Second, we examined performance relative to the cumulative incidence of UNC among all NYC hospitals, following the CMS-endorsed methodology for hospital comparisons.^10^ We used 95% confidence limits from unadjusted models to classify hospitals as performing better, worse, or no different than the overall NYC incidence. We assigned *worse performing* if the lower 95% confidence limit of the hospital UNC estimate was greater than the overall NYC incidence, *better performing* when the upper 95% confidence limit was less than the overall NYC incidence, and *no different* if the confidence interval included the overall NYC incidence.^10,12^ We repeated this exercise for models adjusted for maternal characteristics and identified facilities whose performance designation changed after adjustment. Third, we ranked hospitals from lowest to highest by unadjusted and adjusted UNC rate and examined the distribution of hospital ranking changes after maternal risk adjustment, similar to methods in previous perinatal hospital quality research.^2,13,14,15,16,17^

## Results

Among 254 259 singleton, low-risk births at 37 weeks or later (125 245 female [49.3%] and 129 014 male [50.7%]; 71 768 births [28.2%] to Hispanic, 47 226 births [18.7%] to non-Hispanic Asian, 42 682 births [16.8%] to non-Hispanic Black, and 89 845 births [35.3%] to non-Hispanic White mothers and 2738 births [1.0%] to mothers with another race or ethnicity), 148 393 births (58.4%) were insured by Medicaid (vs 101 633 births [40.0%] with commercial, 2226 births [0.9%] with other insurance, and 2007 births [0.8% ] with no insurance). In our cohort, 104 644 of 253 320 birthing individuals with BMI data (41.3%) had obesity or overweight (BMI ≥25), 26 365 individuals (10.4%) had hypertensive disorders, 26 233 individuals (10.3%) had diabetes, and 59 915 individuals (23.6%) had another chronic condition. Among 39 hospitals, 10 had a level 4 and 23 had a level 3 neonatal intensive care unit (NICU). The NICU level was less than 3 in 6 hospitals (0 hospitals with level 1 and 6 hospitals with level 2). The 2016 to 2018 cumulative incidence of UNCs in NYC hospitals was 37.1 UNCs per 1000 births. Of these, 11.5 UNCs per 1000 births were severe complications and 25.6 UNCs per 1000 births were moderate complications (Table 1). Infants of mothers with preadmission risk factors had increased UNC risk. For example, UNC rates per 1000 births were 104.4 UNCs vs 35.8 UNCs comparing infants of individuals with and without preeclampsia and 91.9 UNCs vs 36.5 UNCs for infants of individuals with vs without preexisting diabetes.

**Table 1.** Cumulative Incidence of UNCs by Sociodemographic, Clinical, and Hospital Characteristic

Characteristic	Births, No. (%)^a^	UNCs, No./1000 births^b^		
		Severe	Moderate	Total
Overall	254 259 (100)	11.5	25.6	37.1
Race and ethnicity^c^				
Hispanic	71 768 (28.2)	13.3	28.8	42.1
Non-Hispanic Asian	47 226 (18.6)	10.1	24.1	34.2
Non-Hispanic Black	42 682 (16.8)	17.2	34.8	52.0
Non-Hispanic White	89 845 (35.3)	8.2	19.3	27.6
Other	2738 (1.0)	12.1	25.9	38.0
Maternal age, y				
<20	6693 (2.6)	22.4	37.7	60.1
20-34	182 941 (72.0)	11.7	25.4	37.1
≥35	64 625 (25.4)	9.9	24.9	34.8
Maternal education (n = 253 590)				
<High school	41 168 (16.2)	13.0	30.4	43.4
High school diploma or GED	56 973 (22.5)	12.1	27.4	39.5
>High school	155 449 (61.3)	10.9	23.5	34.4
Insurance coverage				
Medicaid	148 393 (58.4)	12.4	28.8	41.2
Private insurance	101 633 (40.0)	10.0	20.6	30.6
Other payor	2226 (0.9)	21.6	36.4	58.0
Uninsured	2007 (0.8)	17.4	26.4	43.8
Parity (n = 254 200)				
Nulliparous	109 527 (43.1)	16.7	32.6	49.3
Multiparous	144 673 (56.9)	7.6	20.3	20.5
Previous cesarean				
Yes	12 160 (4.8)	12.1	37.3	49.3
No	242 099 (95.2)	11.5	25.0	36.5
Late prenatal care entry (n = 249 998)				
Yes	59 328 (23.7)	14.0	32.1	46.0
No	190 670 (76.3)	10.7	23.4	34.0
Prepregnancy BMI (n = 253 320)^d^				
Underweight	13 418 (5.3)	9.5	20.5	30.0
Normal weight	135 258 (53.4)	9.8	22.1	31.9
Overweight	62 641 (24.7)	12.2	28.5	40.6
Class I-II obesity	36 866 (14.6)	16.2	32.5	48.7
Class III obesity	5137 (2.0)	20.2	41.9	62.1
Preeclampsia				
Yes	4886 (1.9)	27.6	76.7	104.4
No	249 373 (98.1)	11.2	24.6	35.8
Gestational hypertension				
Yes	16 298 (6.4)	18.6	44.7	63.3
No	237 961 (93.6)	11.1	24.3	35.3
Chronic hypertension				
Yes	5181 (2.0)	16.4	55.8	72.2
No	249 078 (98.0)	11.4	24.9	36.4
Preexisting diabetes				
Yes	2578 (1.0)	27.9	64.0	91.9
No	251 681 (99.0)	11.4	25.2	36.5
Gestational diabetes				
Yes	23 655 (9.3)	14.5	36.0	50.5
No	230 604 (90.7)	11.2	24.5	35.7
Other maternal comorbidity^e^				
Yes	59 915 (23.6)	14.5	35.0	49.5
No	194 334 (76.4)	10.6	22.7	33.3
Gestational age, wk				
Early term (37-38)	60 741 (23.9)	12.1	31.2	43.3
Full term (39-40)	167 032 (65.7)	10.9	23.0	33.8
Late term (41)	25 252 (9.9)	14.5	28.8	43.3
Postterm (≥42)	1234 (0.5)	13.8	33,2	47.0
Mode of delivery				
Vaginal	179 060 (70.4)	10.0	22.4	32.4
Cesarean	75 195 (29.6)	15.3	33.0	48.4
Incidence by characteristic of 39 delivery hospitals				
Annual delivery volume by quartile, No. deliveries				
Low (344 to <601)	17 464 (6.9)	22.6	41.2	63.7
Medium (601 to <954)	36 154 (14.2)	17.0	31.2	48.3
High (954 to <1663)	66 300 (26.1)	12.3	32.8	45.1
Very high (≥1663)	134 341 (52.8)	8.2	18.4	26.7
Medicaid deliveries, quartile, %				
First (<45.70)	105 401 (41.5)	10.1	20.5	30.6
Second (45.70-81.22)	71 728 (28.2)	11.0	23.6	34.5
Third (81.22-90.72)	46 302 (18.2)	12.8	30.5	43.3
Fourth (≥90.72)	30 828 (12.1)	15.9	40.2	56.1
Births to Black mothers, quartile, %				
First (<7.39)	91 064 35.8)	9.1	23.1	32.2
Second (7.39-<14.22)	70 297 (27.7)	9.7	21.0	30.8
Third (14.22-32.83)	63 390 (24.9)	13.2	28.8	42.0
Fourth (≥32.83)	29 508 (11.6)	19.7	37.0	56.7
Births to Hispanic mothers, quartile, %				
First (<16.67)	81 359 (32.0)	11.8	23.0	34.8
Second (16.67-25.71)	85 345 (33.6)	8.8	21.4	30.2
Third (25.71-56.25)	56 579 (22.3)	12.4	27.8	40.2
Fourth (≥56.25)	30 976 (12.2)	16.8	39.8	56.6
Hospital ownership				
Public (12 hospitals)	36 200 (14.2)	16.5	38.3	54.8
Private (27 hospitals)	218 059 (85.8)	10.7	23.5	34.2

Abbreviations: BMI, body mass index (calculated as weight in kilograms divided by height in meters squared); GED, General Educational Development test; UNC, unexpected neonatal complication.

^a^ There were 59 missing observations (<0.1%) for parity, 4261 missing observations (1.7%) for prenatal care entry, 669 missing observations (0.3%) for education, and 939 missing observations (0.4%) for BMI. There were no missing data for other characteristics listed in the table.

^b^ UNC rates are given among 254 259 newborns delivered at 37 weeks or later in New York City hospitals in 2016 to 2018.

^c^ Maternal race and ethnicity was self-reported on the birth certificate. Hispanic ethnicity includes Cuban, Mexican, Puerto Rican, or other Hispanic origin. Asian race includes Asian Indian, Chinese, Filipino, Japanese, Korean, Vietnamese, or other (not specified) Asian. The category for other race includes American Indian or Alaska Native, Guamanian or Chamorro, Native Hawaiian, Other Pacific Islander, Samoan, and other (not specified) race.

^d^ BMI categories were defined as underweight (<18.5), normal weight (18.5 to <25), overweight (25 to <30), class 1 to 2 obesity (30 to <40), and class 3 obesity (≥40).

^e^ Includes diagnoses in the following categories: asthma, anemia, bariatric surgery, thyrotoxicosis, and cardiac, pulmonary, kidney, bleeding, digestive, autoimmune, and major mental health disorders.

### Hospital Complication Rates

**Figure 1.**
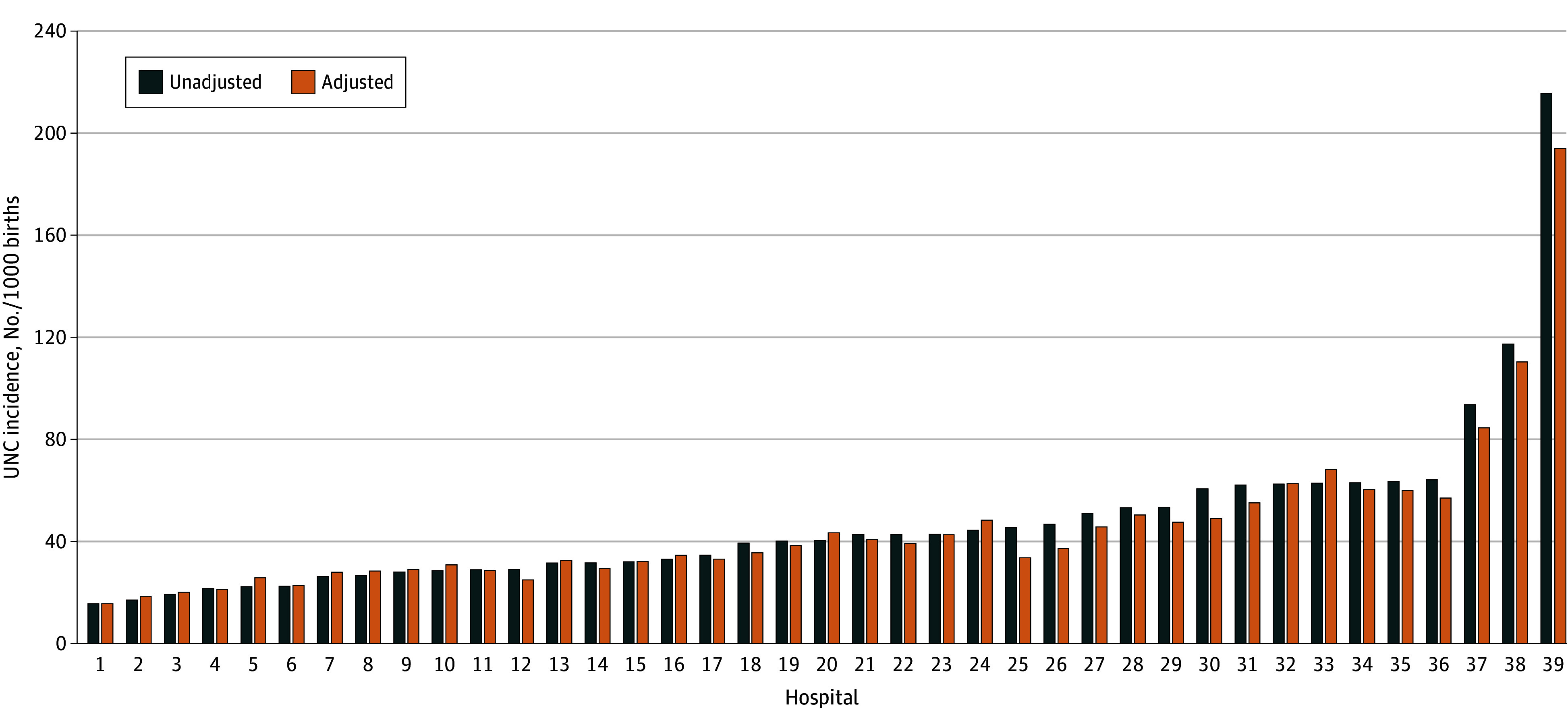
Unexpected Newborn Complication (UNC) Rates Among 39 Hospitals Rates are given among 39 New York City hospitals from 2016 to 2018. Adjusted models include parity, previous cesarean delivery, late entry into prenatal care, maternal age, educational attainment, insurance coverage, prepregnancy body mass index (calculated as weight in kilograms divided by height in meters squared), preeclampsia, gestational hypertension, chronic hypertension, gestational diabetes, preexisting diabetes, pulmonary hypertension, asthma, pulmonary disease, bleeding disease, kidney disease, autoimmune condition, substance use disorder, anemia, bariatric surgery, major mental health disorder, neuromuscular disorder, and thyrotoxicosis.

Unadjusted hospital UNC rates ranged from 15.6 to 215.5 UNCs per 1000 births (Figure 1). Hospitals with high complication rates generally had lower delivery volumes and higher proportions of births to individuals with Medicaid, Black or Hispanic people, and individuals with medical comorbidities (Table 1). Hospital UNC rates ranged from 15.6 to 194.0 UNCs per 1000 births when adjusted for maternal characteristics. The median (IQR) hospital change in UNC after adjustment was −1.4 (−4.7 to 1.0) complications per 1000 births. Patterns in our results were qualitatively similar when we used the CMS mixed-effects regression approach to model hospital performance (eFigure 2 in Supplement 1), although there was less variability in UNC estimates and a smaller median change with adjustment using this method.

We examined whether the median change in hospital UNC rates from risk adjustment differed by facility characteristics (Table 2). The median (IQR) change per 1000 births for adjusted vs unadjusted rates showed that hospitals with low (<601 deliveries/year; −2.8 [−7.0 to −1.6] UNCs) and medium (601 to <954 deliveries/year; −3.9 [−7.1 to −1.9] UNCs) volume, public hospitals (−3.6 [−6.2 to −2.3] UNCs), and those with high proportions of births covered by Medicaid (eg, ≥90.72%; −3.7 [−5.3 to −1.9] UNCs) or to Black (eg, ≥32.83%; −5.3 [−9.1 to −2.2] UNCs) or Hispanic (eg, ≥6.25%; −3.7 [−5.3 to −1.9] UNCs) birthing people had significantly lower UNC rates (improved performance) after adjustment. Conversely, hospitals with very high delivery volume and those with the lowest proportions of Black, Hispanic, and Medicaid births and private ownership had higher rates (worse performance) or no change in complication rates after adjustment.

**Table 2.** Median Change in Hospital UNC Rate by Hospital Characteristic

Characteristic	UNCs, median (IQR), No./1000 births			*P* value for median change
	Unadjusted rate	Adjusted rate^a^	Change	
Annual delivery volume, quartile, No. deliveries				
Low (<601)	53.2 (39.3 to 62.5)	50.4 (35.6- to 62.6)	−2.8 (−7.0 to −1.6)	.02
Medium (601 to <954)	44.0 (29.1 to 63.5)	40.0 (24.9 to 57.0)	−3.9 (−7.1 to −1.9)	.01
High (954 to <1663)	45.50 (32.0 to 60.6)	45.1 (32.0 to 49.0)	−0.3 (−5.9 to 1.8)	.38
Very high (≥1663)	27.2 (22.3 to 31.5)	28.4 (22.7 to 32.6)	1.0 (0.3 to 2.4))	.04
Medicaid deliveries, quartile, %				
First (<45.70)	31.5 (26.6 to 34.6)	32.0 (28.4 to 33.1)	0.9 (0.0 to 1.8)	.10
Second (45.70 to <81.22)	28.9 (19.2 to 44.4)	30.8 (20.1 to 42.6)	0.0 (−3.5 to 1.5)	.90
Third (81.22 to <90.72)	56.9 (40.1 to 63.5)	49.7 (33.5 to 57.0)	−5.3 (−9.1 to −2.2)	.01
Fourth (≥90.72)	51.0 (39.3 to 62.5)	45.7 (35.6 to 60.3)	−3.7 (−5.3 to −1.9)	.02
Births to Black women, quartile, %				
First (<7.39)	31.8 (22.3 to 42.8)	32.3 (25.8 to 43.4)	1.0 (−0.2 to 1.5)	.43
Second (7.39 to <14.22)	33.0 (22.4 to 40.1)	33.1 (22.7 to 38.4)	0.3 (−0.3 to 1.8)	.30
Third (14.22 to <32.83%)	48.8 (29.1 to 62.5)	41.4 (30.8 to 59.9)	−3.9 (−7.1 to 0.1))	.03
Fourth (≥32.83)	49.3 (42.7 to 64.2)	45.6 (33.5 to 57.0)	−5.3 (−9.1 to −2.2)	.002
Births to Hispanic women, quartile, %				
First (<16.67)	31.8 (28.4 to 62.1)	32.3 (28.6 to 55.1)	0.6 (−7.0 to 2.4)	.92
Second (16.67 to <25.71)	30.5 (22.4 to 44.4)	31.7 (22.7 to 48.3)	1.2 (−0.3 to 1.5))	.27
Third (25.71 to <56.25)	44.1 (31.6 to 60.6)	35.4 (29.3 to 49.0)	−2.9 (−9.5 to −0.2)	.01
Fourth (≥6.25)	51.0 (40.1 to 62.5)	45.7 (38.4 to 60.3)	−3.7 (−5.3 to −1.9)	.008
Hospital ownership				
Public	57.7 (42.7 to 63.3)	52.7 (39.9 to 60.1)	−3.6 (−6.2 to −2.3)	.002
Private	32.0 (26.3 to 45.4)	32.6 (27.9 to 43.4)	−0.5 (−3.7 to 1.5))	.50

Abbreviation: UNC, unexpected newborn complication.

^a^ Adjusted for parity, previous cesarean delivery, late entry into prenatal care, maternal age, educational attainment, insurance coverage, prepregnancy body mass index (calculated as weight in kilograms divided by height in meters squared), preeclampsia, gestational hypertension, chronic hypertension, gestational diabetes, preexisting diabetes, pulmonary hypertension, asthma, pulmonary disease, bleeding disease, kidney disease, autoimmune condition, substance use disorder, anemia, bariatric surgery, major mental health disorder, neuromuscular disorder, and thyrotoxicosis.

### Profiling Analyses

**Figure 2.**
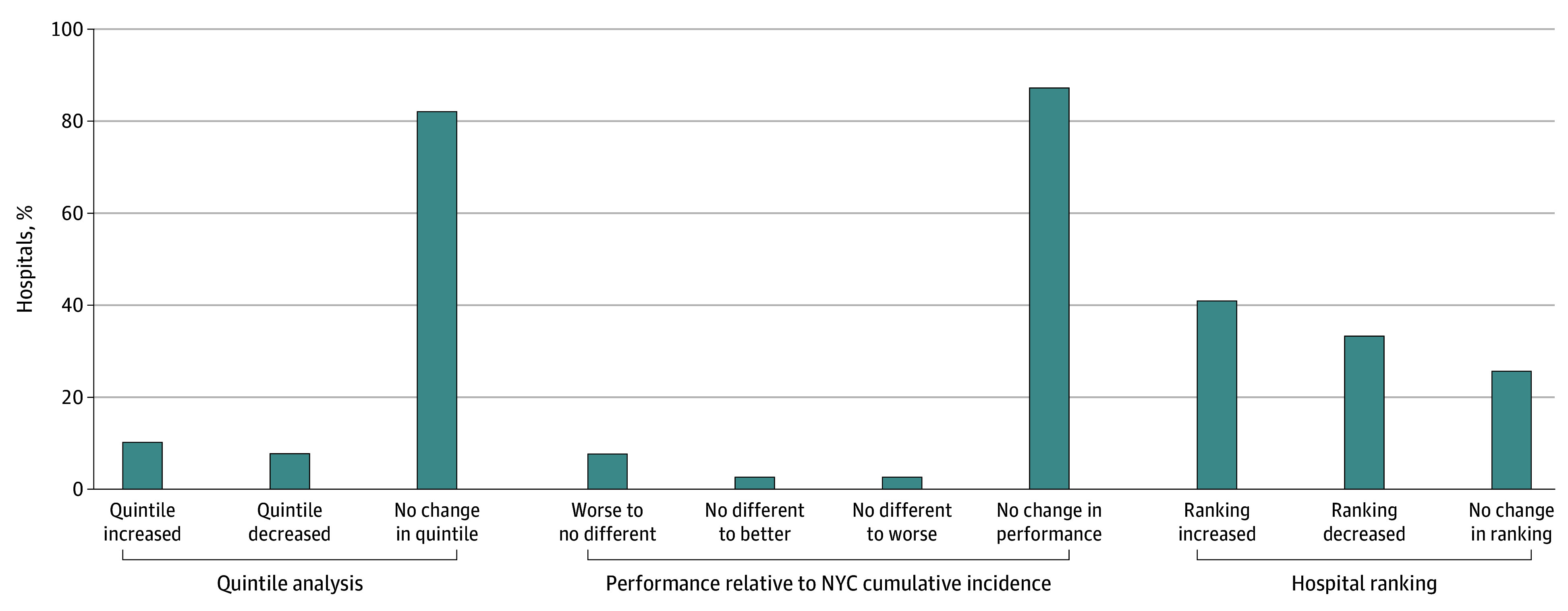
Unadjusted vs Adjusted Hospital Performance on Unexpected Complications Measure Hospital profiling analyses compared unadjusted with adjusted unexpected newborn complication performance. The quintile analysis was based on the *U.S. News & World Report* PC-06 hospital scoring approach.^11^ Performance relative to the New York City (NYC) cumulative incidence was based on Centers for Medicare & Medicaid Services profiling procedures.^12^ The hospital ranking analysis was based on a previously published hospital comparison methodology for obstetric and neonatal outcomes.^14,15,16^ Risk-adjusted models include parity, previous cesarean delivery, late entry into prenatal care, maternal age, educational attainment, insurance coverage, prepregnancy body mass index (calculated as weight in kilograms divided by height in meters squared), preeclampsia, gestational hypertension, chronic hypertension, gestational diabetes, preexisting diabetes, pulmonary hypertension, asthma, pulmonary disease, bleeding disease, kidney disease, autoimmune condition, substance use disorder, anemia, bariatric surgery, major mental health disorder, neuromuscular disorder, and thyrotoxicosis.

We considered the association of risk adjustment with potential hospital profiling strategies (Table 3; Figure 2). First, using the U*.S. News & World Report* quintile scoring system, we found that 7 hospitals (17.9%) shifted 1 quintile comparing risk-adjusted with unadjusted quintile rankings. Of these, 4 hospitals (10.3%) ranked in a higher quintile (worse performance) when using maternal risk–adjusted compared with unadjusted rates and 3 hospitals (7.7%) moved to a lower quintile with adjustment (improved performance). In a second profiling analysis comparing hospital performance relative to the overall NYC incidence (CMS methodology), approximately 5 hospitals (12.8%) changed performance rating after adjustment. Of these, 4 hospitals (10.3%) improved their rating (moving from worse than the overall NYC incidence to no different than the NYC incidence or from no different to better than the NYC incidence) and 1 hospital (2.6%) declined from a rating of no different to worse than the NYC incidence (individual hospital changes are shown in eFigure 3 in Supplement 1). Finally, in our assessment of individual hospital rankings, we found that 29 hospitals (74.4%) changed ranking comparing unadjusted with adjusted UNC performance, with a median (IQR) change of 0 (−1 to 6) positions and a range of −8 to 4 positions.

**Table 3.** Hospital Profiling Analyses Comparing Unadjusted and Adjusted Hospital Performance on UNC Measure^a^

Method	Description
Quintile analysis	Based on* U.S. News & World Report* PC-06 hospital scoring approach^11^ Calculated quintiles of UNCs among NYC hospitals. Examined changes in quintile with adjustment of UNC rates
Performance relative to 2016-2018 NYC cumulative incidence	Based on CMS profiling procedures^12^ Compared unadjusted hospital UNC with UNC among all NYC hospital births (37.1 UNCs/1000 births). If LCL >37.1, then hospital = worse performing; if UCL <37.1, then hospital = better performing. If CL included 37.1, then hospital = no different performance than overall NYC incidence. Repeated this comparison using models adjusted for maternal characteristics and identified facilities whose performance designation changed after adjustment
Hospital rankings	Based on previously published hospital comparison methodology for obstetric and neonatal outcomes^14,15,16^ Ranked hospitals from 1 (lowest UNC) to 39 (highest UNC) and examined ranking changes after adjustment

Abbreviations: CMS, Centers for Medicare & Medicaid Services; LCL, lower confidence limit; NYC, New York City; UCL, upper confidence limit; UNC, unexpected newborn complications.

^a^ Risk-adjusted models included parity, previous cesarean delivery, late entry into prenatal care, maternal age, educational attainment, insurance coverage, prepregnancy body mass index (calculated as weight in kilograms divided by height in meters squared), preeclampsia, gestational hypertension, chronic hypertension, gestational diabetes, preexisting diabetes, pulmonary hypertension, asthma, pulmonary disease, bleeding disease, kidney disease, autoimmune condition, substance use disorder, anemia, bariatric surgery, major mental health disorder, neuromuscular disorder, and thyrotoxicosis.

## Discussion

In this cohort study, we observed wide variation among NYC hospitals in unadjusted rates of unexpected complications among newborns who were born at 37 weeks or later, a Joint Commission-endorsed measure of perinatal care quality. Despite associations between maternal characteristics and UNC rates, the overall magnitude of change associated with maternal risk adjustment was modest. We observed patterns in the association between risk adjustment and UNC by hospital characteristic, with significantly improved rates (lower UNC rates) after adjustment among public hospitals, hospitals with lower delivery volume, and those with higher proportions of births covered by Medicaid or to Black or Hispanic birthing people.

Accounting for patient attributes may facilitate fair comparisons by identifying hospital performance variation that reflects differences in care as opposed to differences in the medical acuity of its patient population.^10^ Maternal health and social characteristics are associated with newborn outcomes, including respiratory morbidity, preterm birth, low birthweight, and NICU admission,^18,19,20,21,22,23,24^ and have been specifically associated with morbidity among newborns at low-risk born at 37 weeks or later in prior studies of this UNC perinatal quality measure.^16,17,25^ We confirmed individual-level associations in our data and that controlling for these factors was associated with modest changes in hospital UNC rates. Patient case mix explained little hospital-level UNC variation in previous studies using administrative data from Florida births^17^ and a national sample of US vital records.^25^ We expand on previous work by demonstrating that the association of maternal risk adjustment with UNC rates was not universal across hospital types and that changes in outcomes were greater among hospitals with large Black, Hispanic, and Medicaid-insured populations in our setting. Critically, although PC-06 is not currently used for payment incentives or penalties, evaluating hospital performance without accounting for maternal risk could impact reputation and withhold resources from hospitals serving populations with the greatest medical and social need. This practice could have the unintended consequence of limiting hospital capacity for quality improvement and social support interventions, potentially exacerbating disparities. Our study provides evidence that may be useful in refining PC-06 reporting criteria. For example, examining hospital performance relative to facilities with similar characteristics (eg, delivery volume) or including individual-level risk adjustment for patient health at admission may improve identification of unwarranted variation attributable to differences in quality of care.

Our findings suggest that even modest changes in hospital UNC rates with maternal risk adjustment may be associated with results of hospital profiling on obstetric and neonatal care quality. PC-06 is currently used in national benchmarking by publications, such as the *U.S. News & World Report* Best Hospitals for Maternity Care.^11^ When we computed quintile rankings to summarize UNC performance among NYC hospitals, we found that roughly 18% of hospitals changed quintiles and 8% had an improved quintile ranking comparing adjusted with unadjusted approaches. If results of a similar magnitude were extrapolated to a national level, then including maternal risk adjustment in PC-06 reporting criteria would be associated with changes for 117 (52 positive, 65 negative) of 649 hospitals that responded to the 2022 *U.S. News & World Report* nationwide maternity survey. Using a CMS hospital comparison method, roughly 10% of hospitals had improved performance relative to the NYC incidence (moving from worse to no difference in performance or from no difference to better performance) when using adjusted as opposed to unadjusted rates. These exercises suggest that maternal risk adjustment may be associated with hospital comparisons that inform where patients choose to deliver and where clinicians choose to practice.

The practical complexity of hospital reporting is a critical consideration in determining optimal approaches to risk adjustment of quality measures. The PC-06 *ICD-10* algorithm requires hospitals to abstract a very large number of data elements, with roughly 600 codes for denominator inclusions and exclusions and 250 diagnosis and procedure codes for numerator specifications. Complications are further divided by severity and into 6 subcategories by diagnosis type. PC-06 was developed for use with infant discharge data, and we acknowledge the limited availability and logistical challenges of working with linked maternal-infant data. Still, our analyses demonstrate the limitations of a siloed approach to quality measurement, particularly for hospitals with the worst morbidity outcomes and those disproportionately serving Black and Hispanic patients and individuals with low income. Therefore, implementing maternal risk adjustment requires weighing the cost-benefit of additional reporting burden, including time demands and potential threats to accuracy, and our analyses contribute initial evidence to consider in PC-06 refinement. Next research steps should address analytic options for risk adjustment (eg, identifying a minimally sufficient set of maternal risk factors, using validated obstetric risk scores,^7,26,27^ or adding maternal conditions to denominator restrictions) and implementation research with hospital staff on reporting challenges and opportunities for improvement. The Joint Commission will add a new electronic clinical quality measure, ePC-07 Severe Obstetric Complications, in 2024.^28^ This measure will include risk adjustment using *ICD-10* codes for conditions present on delivery admission, which may demonstrate feasibility for replication in UNC measurement.

Finally, we previously asserted the importance of a maternal-infant dyadic approach to perinatal quality improvement.^29^ We showed that, among very preterm deliveries in NYC, Black and Hispanic individuals disproportionately delivered at hospitals with worse outcomes for the pregnant person and newborn,^30^ highlighting the importance of integrating and strengthening quality improvement for high-risk obstetrics and neonatal care. Individual-level associations and hospital rates found in this study support pursuing policy and clinical approaches for the maternal-infant dyad among relatively low-risk births at 37 weeks or later to evaluate obstetric and neonatal quality jointly and address shared underlying factors associated with preventable adverse outcomes.

### Limitations and Strengths

This study has several limitations. Our study may be limited by the accuracy of diagnosis and procedure codes in administrative data and variation in coding practices across facilities. We used birth certificates and discharge abstracts for conditions such as maternal comorbidities to improve validity.^24^ The PC-06 algorithm also includes protections against undercoding and overcoding in billing data. Reliability of estimates for this rare outcome may be limited in low-volume hospitals, but no hospitals had an annual delivery volume of fewer than 300 births, which is the threshold recommendation for stable UNC estimates. We also conducted a sensitivity analysis using the CMS method of risk standardization, which stabilizes estimates from facilities with few events, and observed similar patterns in our results. To protect hospital confidentiality, we did not present complication rates by NICU level given the small sample of hospitals with an NICU level less than 3 (0 hospitals with level 1 and 6 hospitals with level 2). Our results represent births in a diverse, densely populated urban setting and may not generalize to all US regions. Additionally, we did not evaluate hospital characteristics in profiling analyses given the small sample size of hospitals whose performance rating changed. Strengths of our study include analysis of a racially and ethnically diverse population of all birthing people with live births. We present novel findings on the implications of risk adjustment for hospital profiling, using methods currently used in national comparisons of hospital care.

## Conclusions

Individual-level associations found in this cohort study underscore the importance of maternal risk assessment for improvement in outcomes in even relatively routine births. Adjustment for case mix was associated with modest overall changes in hospital UNC rates. PC-06 is a new measure, and our results suggest that decisions regarding its use for hospital benchmarking or profiling should consider implications of small changes in rates, particularly for lower-volume or public hospitals or those with substantial populations of Medicaid-insured patients and Hispanic and Black patients.

## Notes

Supplement 1. **eFigure 1.** Study Population Flow Diagram **eTable 1.** Classification of Unexpected Newborn Complications According to *ICD-10* Diagnosis and Procedure Codes **eTable 2.** Ascertainment of Maternal Comorbidities From Hospital Discharge Record (*ICD-10-CM*) or Vital Statistics **eFigure 2.** Unexpected Newborn Complications Among 39 New York City Hospitals, Using CMS Hospital Profiling Methodology for Risk Standardization **eFigure 3.** Unadjusted and Adjusted (95% CI) Unexpected Newborn Complication Rate, per 1000 Births, in 39 New York City Hospitals, 2016-2018

Supplement 2.
Data Sharing Statement
