# Supplementary material for: Maternal Characteristics and Rates of Unexpected Complications in Term Newborns by Hospital
Source: JAMA Netw Open. 2024 May 20;7(5):e2411699. doi: 10.1001/jamanetworkopen.2024.11699 (PMC11107302; doi:10.1001/jamanetworkopen.2024.11699)
Supplement: Supplement 1. — eFigure 1. Study Population Flow Diagram eTable 1. Classification of Unexpected Newborn Complications According to ICD-10 Diagnosis and Procedure Codes eTable 2. Ascertainment of Maternal Comorbidities From Hospital Discharge Record (ICD-10-CM) or Vital Statistics eFigure 2. Unexpected Newborn Complications Among 39 New York City Hospitals, Using CMS Hospital Profiling Methodology for Risk Standardization eFigure 3. Unadjusted and Adjusted (95% CI) Unexpected Newborn Complication Rate, per 1000 Births, in 39 New York City Hospitals, 2016-2018 [file jamanetwopen-e2411699-s001.pdf]

Supplemental Online Content

Glazer KB, Zeitlin J, Boychuk N, et al. Maternal characteristics and rates of unexpected complications in term newborns by hospital. *JAMA Netw Open*. 2024;7(5):e2411699. doi:10.1001/jamanetworkopen.2024.11699

eFigure 1. Study Population Flow Diagram

eTable 1. Classification of Unexpected Newborn Complications According to *ICD-10* Diagnosis and Procedure Codes

eTable 2. Ascertainment of Maternal Comorbidities From Hospital Discharge Record (*ICD-10-CM*) or Vital Statistics

eFigure 2. Unexpected Newborn Complications Among 39 New York City Hospitals, Using CMS Hospital Profiling Methodology for Risk Standardization

eFigure 3. Unadjusted and Adjusted (95% CI) Unexpected Newborn Complication Rate, per 1000 Births, in 39 New York City Hospitals, 2016-2018

This supplemental material has been provided by the authors to give readers additional information about their work.

© 2024 Glazer KB et al. *JAMA Network Open.*

Supplemental Figure1. Study population flow diagram

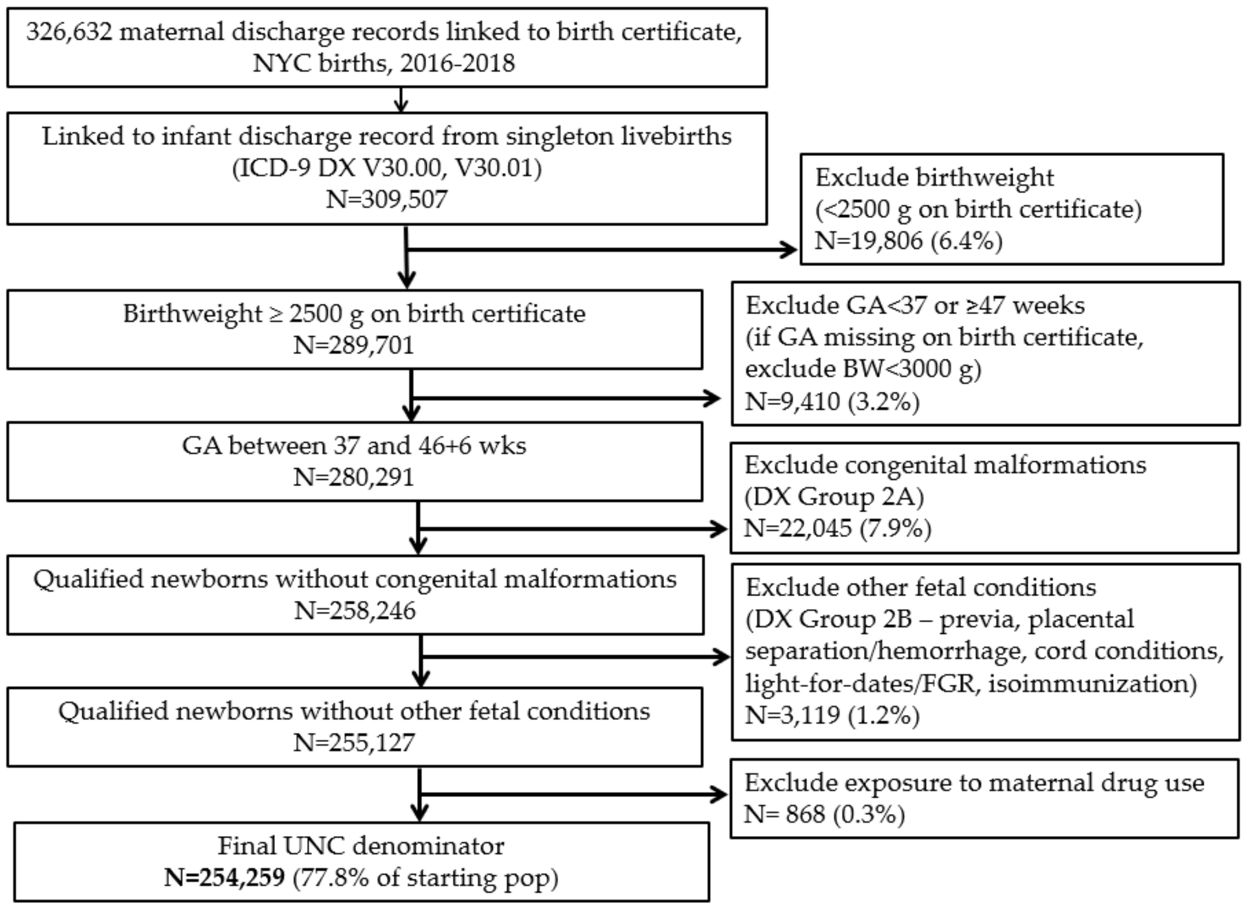

© 2024 Glazer KB et al. *JAMA Network Open.*

Supplemental Table 1. Classification of unexpected newborn complications according to ICD-10 diagnosis and procedure codes

ICD-10 code	Shortened Description
**TJC Table Name (Number): Severe Birth Trauma (11.36)**	
P100	Subdural hemorrhage due to birth injury
P101	Cerebral hemorrhage due to birth injury
P102	Intraventricular hemorrhage due to birth injury
P103	Subarachnoid hemorrhage due to birth injury
P104	Tentorial tear due to birth injury
P108	Other intracranial lacerations and hemorrhages due to birth injury
P109	Unspecified intracranial laceration and hemorrhage due to birth injury
P110	Cerebral edema due to birth injury
P111	Other specified brain damage due to birth injury
P112	Unspecified brain damage due to birth injury
P114	Birth injury to other cranial nerves
P115	Birth injury to spine and spinal cord
P119	Birth injury to central nervous system, unspecified
P122	Epicranial subaponeurotic hemorrhage due to birth injury
P130	Fracture of skull due to birth injury
P131	Other birth injuries to skull
P132	Birth injury to femur
P133	Birth injury to other long bones
P140	Erb's paralysis due to birth injury
P141	Klumpke's paralysis due to birth injury
P142	Phrenic nerve paralysis due to birth injury
P143	Other brachial plexus birth injuries
P148	Birth injuries to other parts of peripheral nervous system
P149	Birth injury to peripheral nervous system, unspecified
P150	Birth injury to liver
P151	Birth injury to spleen
P510	Massive umbilical hemorrhage of newborn
**Severe Hypoxia/Asphyxia (11.37)**	
G8110	Spastic hemiplegia affecting unspecified side
G8111	Spastic hemiplegia affecting right dominant side
G8112	Spastic hemiplegia affecting left dominant side
G8113	Spastic hemiplegia affecting right nondominant side
G8114	Spastic hemiplegia affecting left nondominant side
G931	Anoxic brain damage, not elsewhere classified
P910	Neonatal cerebral ischemia
P912	Neonatal cerebral leukomalacia
P914	Neonatal cerebral depression
P915	Neonatal coma
P9160	Hypoxic ischemic encephalopathy [HIE], unspecified
P9161	Mild hypoxic ischemic encephalopathy [HIE]
P9162	Moderate hypoxic ischemic encephalopathy [HIE]
P9163	Severe hypoxic ischemic encephalopathy [HIE]
R0901	Asphyxia
R0902	Hypoxemia
**Severe Shock and Resuscitation (11.38)**	
I469	Cardiac arrest, cause unspecified
N170	Acute kidney failure with tubular necrosis
N178	Other acute kidney failure
N179	Acute kidney failure, unspecified
P290	Neonatal cardiac failure
P294	Transient myocardial ischemia in newborn
P2981	Cardiac arrest of newborn
P60	Disseminated intravascular coagulation of newborn
P771	Stage 1 necrotizing enterocolitis in newborn
P772	Stage 2 necrotizing enterocolitis in newborn
P773	Stage 3 necrotizing enterocolitis in newborn
P779	Necrotizing enterocolitis in newborn, unspecified
R570	Cardiogenic shock
R571	Hypovolemic shock
R578	Other shock
R579	Shock, unspecified
**Neonatal Severe Respiratory Complications (11.39)**	

© 2024 Glazer KB et al. *JAMA Network Open.*

P260	Tracheobronchial hemorrhage originating in the perinatal period
P261	Massive pulmonary hemorrhage originating in the perinatal period
P268	Other pulmonary hemorrhages originating in the perinatal period
P269	Unspecified pulmonary hemorrhage originating in the perinatal period
**Neonatal Severe Infection (11.40)**	
P230	Congenital pneumonia due to viral agent
P231	Congenital pneumonia due to Chlamydia
P232	Congenital pneumonia due to staphylococcus
P233	Congenital pneumonia due to streptococcus, group B
P234	Congenital pneumonia due to Escherichia coli
P235	Congenital pneumonia due to Pseudomonas
P236	Congenital pneumonia due to other bacterial agents
P238	Congenital pneumonia due to other organisms
P239	Congenital pneumonia, unspecified
R6520	Severe sepsis without septic shock
R6521	Severe sepsis with septic shock
**Neonatal Severe Neurological Complications (11.41)**	
G913	Post-traumatic hydrocephalus, unspecified
G9340	Encephalopathy, unspecified
G9341	Metabolic encephalopathy
G9349	Other encephalopathy
G9382	Brain death
P520	Intraventricular (nontraumatic) hemorrhage, grade 1, of newborn
P521	Intraventricular (nontraumatic) hemorrhage, grade 2, of newborn
P5221	Intraventricular (nontraumatic) hemorrhage, grade 3, of newborn
P5222	Intraventricular (nontraumatic) hemorrhage, grade 4, of newborn
P523	Unspecified intraventricular (nontraumatic) hemorrhage of newborn
P524	Intracerebral (nontraumatic) hemorrhage of newborn
P525	Subarachnoid (nontraumatic) hemorrhage of newborn
P526	Cerebellar (nontraumatic) and posterior fossa hemorrhage of newborn
P528	Other intracranial (nontraumatic) hemorrhages of newborn
P529	Intracranial (nontraumatic) hemorrhage of newborn, unspecified
P90	Convulsions of newborn
**Severe Shock and Respiratory Procedures (11.42)**	
03HY02Z	Insertion of Monitoring Device into Upper Artery, Open Approach
03HY03Z	Insertion of Infusion Device into Upper Artery, Open Approach
03HY0DZ	Insertion of Intraluminal Device into Upper Artery, Open Approach
03HY0YZ	Insertion of Other Device into Upper Artery, Open Approach
03HY32Z	Insertion of Monitoring Device into Upper Artery, Percutaneous Approach
03HY33Z	Insertion of Infusion Device into Upper Artery, Percutaneous Approach
03HY3DZ	Insertion of Intraluminal Device into Upper Artery, Percutaneous Approach
03HY3YZ	Insertion of Other Device into Upper Artery, Percutaneous Approach
03HY42Z	Insertion of Monitoring Device into Upper Artery, Percutaneous Endoscopic Approach
03HY43Z	Insertion of Infusion Device into Upper Artery, Percutaneous Endoscopic Approach
03HY4DZ	Insertion of Intraluminal Device into Upper Artery, Percutaneous Endoscopic Approach
03HY4YZ	Insertion of Other Device into Upper Artery, Percutaneous Endoscopic Approach
04HY02Z	Insertion of Monitoring Device into Lower Artery, Open Approach
04HY03Z	Insertion of Infusion Device into Lower Artery, Open Approach
04HY0DZ	Insertion of Intraluminal Device into Lower Artery, Open Approach
04HY32Z	Insertion of Monitoring Device into Lower Artery, Percutaneous Approach
04HY33Z	Insertion of Infusion Device into Lower Artery, Percutaneous Approach
04HY3DZ	Insertion of Intraluminal Device into Lower Artery, Percutaneous Approach
04HY42Z	Insertion of Monitoring Device into Lower Artery, Percutaneous Endoscopic Approach
04HY43Z	Insertion of Infusion Device into Lower Artery, Percutaneous Endoscopic Approach
04HY4DZ	Insertion of Intraluminal Device into Lower Artery, Percutaneous Endoscopic Approach
5A12012	Performance of Cardiac Output, Single, Manual
5A2204Z	Restoration of Cardiac Rhythm, Single
**Neonatal Severe Respiratory Procedures (11.43)**	
0W9930Z	Drainage of Right Pleural Cavity with Drainage Device, Percutaneous Approach
0W993ZZ	Drainage of Right Pleural Cavity, Percutaneous Approach
0W9940Z	Drainage of Right Pleural Cavity with Drainage Device, Percutaneous Endoscopic Approach
0W994ZZ	Drainage of Right Pleural Cavity, Percutaneous Endoscopic Approach
0W9B30Z	Drainage of Left Pleural Cavity with Drainage Device, Percutaneous Approach
0W9B3ZZ	Drainage of Left Pleural Cavity, Percutaneous Approach
0W9B40Z	Drainage of Left Pleural Cavity with Drainage Device, Percutaneous Endoscopic Approach
0W9B4ZZ	Drainage of Left Pleural Cavity, Percutaneous Endoscopic Approach

© 2024 Glazer KB et al. *JAMA Network Open.*

3E0F3SD	Introduction of Nitric Oxide Gas into Respiratory Tract, Percutaneous Approach
3E0F7SD	Introduction of Nitric Oxide Gas into Respiratory Tract, Via Natural or Artificial Opening
3E0F8SD	Introduction of Nitric Oxide Gas into Respiratory Tract, Via Natural or Artificial Opening Endoscopic
5A1522F	Extracorporeal Oxygenation, Membrane, Central
5A1522G	Extracorporeal Oxygenation, Membrane, Peripheral Veno-arterial
5A1522H	Extracorporeal Oxygenation, Membrane, Peripheral Veno-venous
5A15A2F	Extracorporeal Oxygenation, Membrane, Central, Intraoperative
5A15A2G	Extracorporeal Oxygenation, Membrane, Peripheral Veno-arterial, Intraoperative
5A15A2H	Extracorporeal Oxygenation, Membrane, Peripheral Veno-venous, Intraoperative
5A1935Z	Respiratory Ventilation, Less than 24 Consecutive Hours
5A1945Z	Respiratory Ventilation, 24-96 Consecutive Hours
5A1955Z	Respiratory Ventilation, Greater than 96 Consecutive Hours
**Neonatal Severe Neurological Procedures (11.44)**	
0DH63UZ	Insertion of Feeding Device into Stomach, Percutaneous Approach
0DH64UZ	Insertion of Feeding Device into Stomach, Percutaneous Endoscopic Approach
4A0004Z	Measurement of Central Nervous Electrical Activity, Open Approach
4A0034Z	Measurement of Central Nervous Electrical Activity, Percutaneous Approach
4A00X4Z	Measurement of Central Nervous Electrical Activity, External Approach
4A1004Z	Monitoring of Central Nervous Electrical Activity, Open Approach
4A1034Z	Monitoring of Central Nervous Electrical Activity, Percutaneous Approach
4A10X4Z	Monitoring of Central Nervous Electrical Activity, External Approach
6A4Z0ZZ	Hypothermia, Single
6A4Z1ZZ	Hypothermia, Multiple
**Neonatal Severe Septicemia (11.45)**	
P362	Sepsis of newborn due to Staphylococcus aureus
P3630	Sepsis of newborn due to unspecified staphylococci
P3639	Sepsis of newborn due to other staphylococci
P364	Sepsis of newborn due to Escherichia coli
P365	Sepsis of newborn due to anaerobes
P368	Other bacterial sepsis of newborn
P369	Bacterial sepsis of newborn, unspecified
R7881	Bacteremia
**Moderate Birth Trauma (11.46)**	
P113	Birth injury to facial nerve
P138	Birth injuries to other parts of skeleton
P139	Birth injury to skeleton, unspecified
P152	Sternomastoid injury due to birth injury
P155	Birth injury to external genitalia
P156	Subcutaneous fat necrosis due to birth injury
**Moderate Respiratory Complications (11.47)**	
P220	Respiratory distress syndrome of newborn
P250	Interstitial emphysema originating in the perinatal period
P251	Pneumothorax originating in the perinatal period
P252	Pneumomediastinum originating in the perinatal period
P253	Pneumopericardium originating in the perinatal period
P258	Other conditions related to interstitial emphysema originating in the perinatal period
**Moderate Respiratory Complications Procedures (11.48)**	
5A09457	Assistance with Respiratory Ventilation, 24-96 Consecutive Hours, Continuous Positive Airway Pressure
5A09557	Assistance with Respiratory Ventilation, Greater than 96 Consecutive Hours, Continuous Positive Airway Pressure
**Moderate Birth Trauma with LOS (11.49)**	
P024	Newborn affected by prolapsed cord
P030	Newborn affected by breech delivery and extraction
P031	Newborn affected by other malpresentation, malposition and disproportion during labor and delivery
P032	Newborn affected by forceps delivery
P033	Newborn affected by delivery by vacuum extractor [ventouse]
P034	Newborn affected by Cesarean delivery
P040	Newborn affected by maternal anesthesia and analgesia in pregnancy, labor and delivery
P134	Fracture of clavicle due to birth injury
P154	Birth injury to face
P158	Other specified birth injuries
P159	Birth injury, unspecified
P501	Newborn affected by intrauterine (fetal) blood loss from ruptured cord
P518	Other umbilical hemorrhages of newborn
P519	Umbilical hemorrhage of newborn, unspecified
P613	Congenital anemia from fetal blood loss
**Moderate Respiratory Complications with LOS (11.50)**	

P221	Transient tachypnea of newborn
P228	Other respiratory distress of newborn
P229	Respiratory distress of newborn, unspecified
P2401	Meconium aspiration with respiratory symptoms
P2411	Neonatal aspiration of (clear) amniotic fluid and mucus with respiratory symptoms
P2421	Neonatal aspiration of blood with respiratory symptoms
P2481	Other neonatal aspiration with respiratory symptoms
P280	Primary atelectasis of newborn
P2810	Unspecified atelectasis of newborn
P2811	Resorption atelectasis without respiratory distress syndrome
P2819	Other atelectasis of newborn
P282	Cyanotic attacks of newborn
P283	Primary sleep apnea of newborn
P284	Other apnea of newborn
P285	Respiratory failure of newborn
P2881	Respiratory arrest of newborn
P2889	Other specified respiratory conditions of newborn
P289	Respiratory condition of newborn, unspecified
**Moderate Neurological Complications with LOS Procedures (11.51)**	
B02000Z	Computerized Tomography (CT Scan) of Brain using High Osmolar Contrast, Unenhanced and Enhanced
B0200ZZ	Computerized Tomography (CT Scan) of Brain using High Osmolar Contrast
B02010Z	Computerized Tomography (CT Scan) of Brain using Low Osmolar Contrast, Unenhanced and Enhanced
B0201ZZ	Computerized Tomography (CT Scan) of Brain using Low Osmolar Contrast
B020Y0Z	Computerized Tomography (CT Scan) of Brain using Other Contrast, Unenhanced and Enhanced
B020YZZ	Computerized Tomography (CT Scan) of Brain using Other Contrast
B020ZZZ	Computerized Tomography (CT Scan) of Brain
B030Y0Z	Magnetic Resonance Imaging (MRI) of Brain using Other Contrast, Unenhanced and Enhanced
B030YZZ	Magnetic Resonance Imaging (MRI) of Brain using Other Contrast
B030ZZZ	Magnetic Resonance Imaging (MRI) of Brain
B03BY0Z	Magnetic Resonance Imaging (MRI) of Spinal Cord using Other Contrast, Unenhanced and Enhanced
B03BYZZ	Magnetic Resonance Imaging (MRI) of Spinal Cord using Other Contrast
B03BZZZ	Magnetic Resonance Imaging (MRI) of Spinal Cord
BW2800Z	Computerized Tomography (CT Scan) of Head using High Osmolar Contrast, Unenhanced and Enhanced
BW280ZZ	Computerized Tomography (CT Scan) of Head using High Osmolar Contrast
BW2810Z	Computerized Tomography (CT Scan) of Head using Low Osmolar Contrast, Unenhanced and Enhanced
BW281ZZ	Computerized Tomography (CT Scan) of Head using Low Osmolar Contrast
BW28Y0Z	Computerized Tomography (CT Scan) of Head using Other Contrast, Unenhanced and Enhanced
BW28YZZ	Computerized Tomography (CT Scan) of Head using Other Contrast
BW28ZZZ	Computerized Tomography (CT Scan) of Head
BW2900Z	Computerized Tomography (CT Scan) of Head and Neck using High Osmolar Contrast, Unenhanced and Enhanced
BW290ZZ	Computerized Tomography (CT Scan) of Head and Neck using High Osmolar Contrast
BW2910Z	Computerized Tomography (CT Scan) of Head and Neck using Low Osmolar Contrast, Unenhanced and Enhanced
BW291ZZ	Computerized Tomography (CT Scan) of Head and Neck using Low Osmolar Contrast
BW29Y0Z	Computerized Tomography (CT Scan) of Head and Neck using Other Contrast, Unenhanced and Enhanced
BW29YZZ	Computerized Tomography (CT Scan) of Head and Neck using Other Contrast
BW29ZZZ	Computerized Tomography (CT Scan) of Head and Neck
**Moderate Respiratory Complications with LOS Procedures (11.52)**	
5A05121	Extracorporeal Hyperbaric Oxygenation, Intermittent
5A05221	Extracorporeal Hyperbaric Oxygenation, Continuous
5A09357	Assistance with Respiratory Ventilation, Less than 24 Consecutive Hours, Continuous Positive Airway Pressure
5A09358	Assistance with Respiratory Ventilation, Less than 24 Consecutive Hours, Intermittent Positive Airway Pressure
5A09359	Assistance with Respiratory Ventilation, Less than 24 Consecutive Hours, Continuous Negative Airway Pressure
5A0935B	Assistance with Respiratory Ventilation, Less than 24 Consecutive Hours, Intermittent Negative Airway Pressure
5A0935Z	Assistance with Respiratory Ventilation, Less than 24 Consecutive Hours
5A09458	Assistance with Respiratory Ventilation, 24-96 Consecutive Hours, Intermittent Positive Airway Pressure
5A09459	Assistance with Respiratory Ventilation, 24-96 Consecutive Hours, Continuous Negative Airway Pressure
5A0945B	Assistance with Respiratory Ventilation, 24-96 Consecutive Hours, Intermittent Negative Airway Pressure
5A0945Z	Assistance with Respiratory Ventilation, 24-96 Consecutive Hours
5A09558	Assistance with Respiratory Ventilation, Greater than 96 Consecutive Hours, Intermittent Positive Airway Pressure
5A09559	Assistance with Respiratory Ventilation, Greater than 96 Consecutive Hours, Continuous Negative Airway Pressure
5A0955B	Assistance with Respiratory Ventilation, Greater than 96 Consecutive Hours, Intermittent Negative Airway Pressure
5A0955Z	Assistance with Respiratory Ventilation, Greater than 96 Consecutive Hours
**Moderate Infection with LOS (11.53)**	
P0270	Newborn affected by fetal inflammatory response syndrome
P0278	Newborn affected by other conditions from chorioamnionitis
P360	Sepsis of newborn due to streptococcus, group B

P3610	Sepsis of newborn due to unspecified streptococci
P3619	Sepsis of newborn due to other streptococci
P362	Sepsis of newborn due to Staphylococcus aureus
P3630	Sepsis of newborn due to unspecified staphylococci
P3639	Sepsis of newborn due to other staphylococci
P364	Sepsis of newborn due to Escherichia coli
P365	Sepsis of newborn due to anaerobes
P368	Other bacterial sepsis of newborn
P369	Bacterial sepsis of newborn, unspecified
R7881	Bacteremia
**Neonatal Jaundice (11.33)**	
P551	ABO isoimmunization of newborn
P580	Neonatal jaundice due to bruising
P581	Neonatal jaundice due to bleeding
P583	Neonatal jaundice due to polycythemia
P5841	Neonatal jaundice due to drugs or toxins transmitted from mother
P5842	Neonatal jaundice due to drugs or toxins given to newborn
P585	Neonatal jaundice due to swallowed maternal blood
P588	Neonatal jaundice due to other specified excessive hemolysis
P589	Neonatal jaundice due to excessive hemolysis, unspecified
P590	Neonatal jaundice associated with preterm delivery
P591	Inspissated bile syndrome
P5920	Neonatal jaundice from unspecified hepatocellular damage
P5929	Neonatal jaundice from other hepatocellular damage
P593	Neonatal jaundice from breast milk inhibitor
P598	Neonatal jaundice from other specified causes
P599	Neonatal jaundice, unspecified
**Phototherapy (11.34)**	
6A600ZZ	Phototherapy of Skin, Single
6A601ZZ	Phototherapy of Skin, Multiple
**Social Indications (11.35)**	
Z590	Homelessness
Z591	Inadequate housing
Z593	Problems related to living in residential institution
Z595	Extreme poverty
Z598	Other problems related to housing and economic circumstances
Z599	Problem related to housing and economic circumstances, unspecified
Z608	Other problems related to social environment
Z609	Problem related to social environment, unspecified
Z6221	Child in welfare custody

TJC=The Joint Commission, ICD=International Classification of Diseases, LOS=length of stay. From “The Joint Commission Code Tables”, available from California Maternal Quality Care Collaborative, https://www.cmqcc.org/focus-areas/quality-metrics/unexpected-complications-term-newborns.

© 2024 Glazer KB et al. *JAMA Network Open.*

Supplemental Table 2. Ascertainment of maternal comorbidities from hospital discharge record (ICD-10-CM) or vital statistics

Candidate Variable	ICD-10-CM Diagnosis Codes or Vital Statistics (VS)[a]
Preexisting diabetes mellitus	E08x-E13x, O24.0x, O24.1x, O24.3x, O24.8x, Z79.4x or VS
Gestational diabetes mellitus	O24.4x, O24.9x or VS
Preexisting/chronic hypertension	O10x, I10x or VS
Preeclampsia	O11x, O14.0x-O14.2x, O14.9x or VS
Gestational hypertension	O13x or VS
Previous cesarean birth	O34.21x
Pulmonary hypertension	I27.0x, I27.2x
Asthma (acute or moderate/severe)	O99.5x, J45.21x, J45.22x, J45.31x, J45.32x, J45.4x, J45.5x, J45.901x, J45.902x, J45909x, J4599x
Other pulmonary disease	J410x, J411x, J418x, J42x, J430x-J432x, J438x-J441x, J449x, J470x, J471x, J479x, J670x-J679x, J60x-J64x, J66x, J840x-J842x, J848x, J849x, J17x, M3481x, J99x
Bleeding disorder, preexisting	D66x-D69x (excluding 68.8x and 68.9x)
Cardiac disease, preexisting	I05x-I09x, I11x-I13x, I15x, I16x, I20x, I25x, I27.8x, I30x-I39x, I40x, I41x, I44x-I49x, I50.22x, I50.23x, I50.32x, I50.33x, I50.42x, I50.43x, I50.812x, I50.813x, O99.41x, O99.43x O99.42x, Q20x-Q24x, O990.3x
Chronic renal disease	O26.83x, I12x, I13x, N03x, N04x, N05x, N07x, N08x, N11.1x, N11.8x, N11.9x, N18x, N25.0x, N25.1x, N25.81x, N25.89x, N25.9x, N26.9x
Connective tissue or autoimmune disease	M30x-M36x
Placental disorders[a]	O44.03x, O44.13x, O44.23x, O44.33x, O45x, O43.2x
Substance use disorder	F10x-F19x, O99.31x, O99.32x
Anemia, preexisting	O99.01x, O99.02x, D50x, D55x, D56x, D57.1x, D57.20x-D57.40x, D57.80x, D58x, D59x
Bariatric surgery	O99.84x
Digestive disorders	K00x-K09x, K10x-K19x, K20x-K29x, K30x-K39x, K40x-K49x, K50x-K59x, K60x-K69x, K70x-K79x, K80x-K89x, K90x- K95x, O99.6x, O26.6x
Major mental health disorders	O99.34x, F20x-F29x, F30x, F31x-F39x
Social determinants of health	Z55x-Z65x
Neuromuscular diseases	O99.35x, G40x, G70x, G35-G37
Thyrotoxicosis	E05x
Body Mass Index	VS

[a] ICD-10-CM= International Classification of Diseases, Tenth Revision, Clinical Modification; VS=Vital Statistics; "Or VS" indicates condition ascertained as present if identified in either the hospital discharge record or birth certificate; [b]includes placenta previa, complete or partial, placental abruption, and placenta accrete spectrum. Conditions identified in hospital discharge record follow ICD-10 coding algorithm in Leonard SA, Kennedy CJ, Carmichael SL, Lyell DJ, Main EK. An Expanded Obstetric Comorbidity Scoring System for Predicting Severe Maternal Morbidity. Obstet Gynecol. 2020;136(3):440-449, with the following exceptions: We additionally include J45909 (unspecified asthma) and J4599 (exercise-induced bronchospasm) in the category "asthma (moderate or severe)" and G35-G37 (multiple sclerosis) in "neuromuscular diseases" based on input from clinical specialists. We ascertained body mass index based on weight and height self-reported on the birth certificate.

© 2024 Glazer KB et al. *JAMA Network Open.*

Supplemental Figure 2. Unexpected newborn complications among 39 NYC hospitals, using CMS hospital profiling methodology for risk standardization

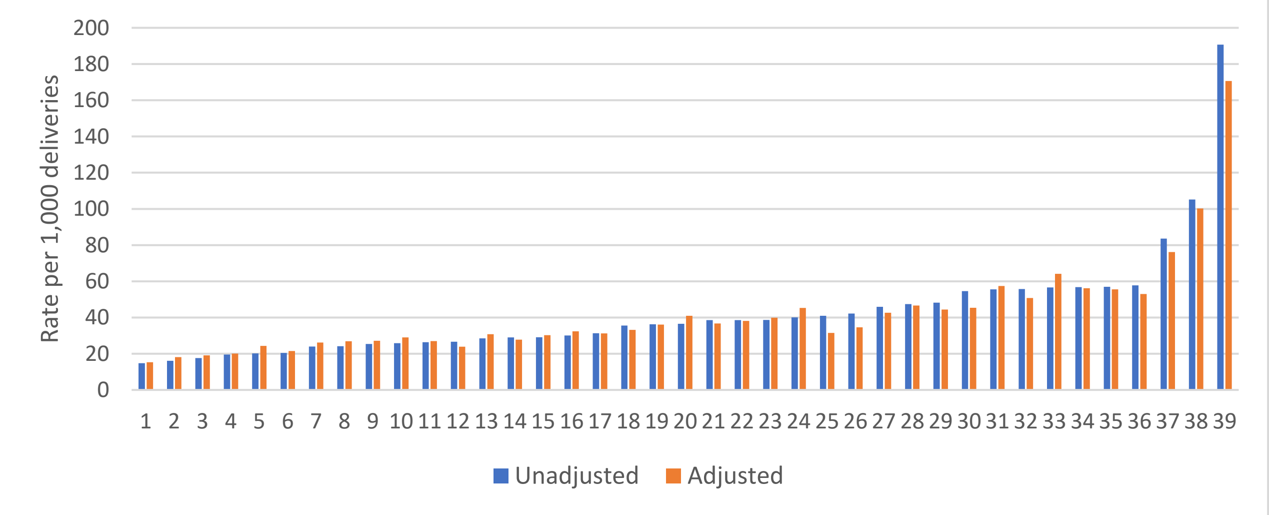

Hospital-specific rates calculated using hierarchical, mixed effects logistic regression, following CMS hospital profiling methodology described in: Ash AS, Fienberg SE, Louis TA, Normand S-LT, Stuken TA, Utts J. Statistical Issues in Assessing Hospital Performance. Committee of Presidents of Statistical Societies. The COPSS-CMS White Paper Committee; 2012. Unadjusted models include hospital random intercept only. Adjusted models include hospital random intercept plus parity, previous cesarean delivery, late entry into prenatal care, maternal age, educational attainment, insurance coverage, prepregnancy BMI, preeclampsia, gestational hypertension, chronic hypertension, gestational diabetes, preexisting diabetes, pulmonary hypertension, asthma, pulmonary disease, bleeding disease, renal disease, autoimmune condition, substance use disorder, anemia, bariatric surgery, major mental health disorder, neuromuscular disorder, and thyrotoxicosis.

Range in unadjusted hospital rates: 14.8 to 190.7/1,000 births
Range in adjusted hospital rates: 15.3 to 170.6/1,000 births
Median [Interquartile Range] change comparing adjusted to unadjusted hospital rates: -0.1 [-3.0 - 2.0]/1000 births

© 2024 Glazer KB et al. *JAMA Network Open.*

Supplemental Figure 3. Unadjusted and adjusted (95% confidence interval) unexpected newborn complication (UNC) rate, per 1000 births, in 39 New York City hospitals, 2016-2018

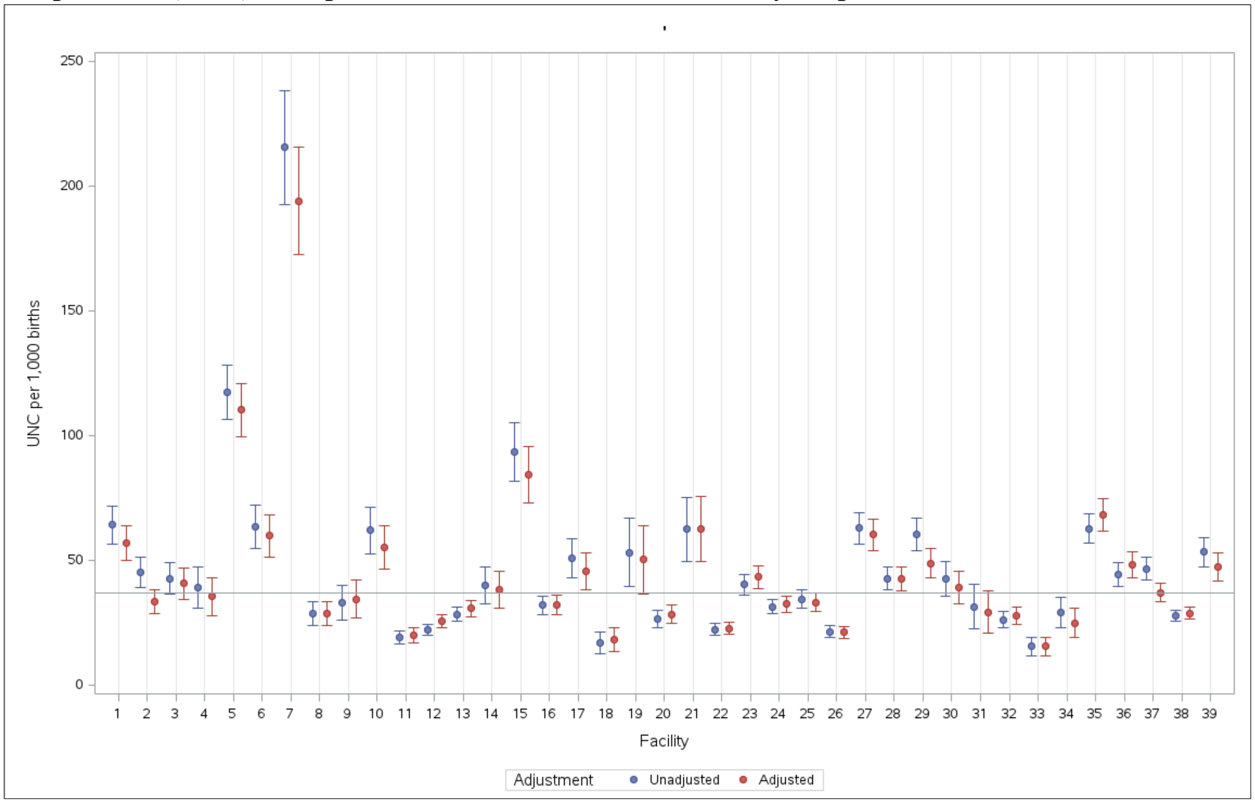

*Horizontal line in plot area indicates the cumulative incidence among all NYC hospital births (37.1 per 1000).

	**Adjusted**			
**Unadjusted**	Worse than NYC	No different than NYC	Better than NYC	Total
Worse than NYC overall incidence	14	3	0	17
No different than NYC overall incidence	1	6	1	8
Better than NYC overall incidence	0	0	14	14
Total	15	9	15	39

Hospitals classified based on performance relative to total UNC incidence among 39 NYC hospitals. Worse performing = lower 95% confidence limit (LCL) of the hospital UNC estimate was greater than the overall NYC incidence; better performing = upper 95% confidence limit (UCL) was less than overall NYC incidence; no different = 95% confidence interval included the overall NYC incidence. Adjusted models included the following covariates: parity, previous cesarean delivery, late entry into prenatal care, maternal age, educational attainment, insurance coverage, pre-pregnancy BMI, preeclampsia, gestational hypertension, chronic hypertension, gestational diabetes, preexisting diabetes, pulmonary hypertension, asthma, pulmonary disease, bleeding disease, renal disease, autoimmune condition, substance use disorder, anemia, bariatric surgery, major mental health disorder, neuromuscular disorder, and thyrotoxicosis.

© 2024 Glazer KB et al. *JAMA Network Open.*
