# Supplementary material for: Maternal Characteristics and Rates of Unexpected Complications in Term Newborns by Hospital
Source: JAMA Netw Open. 2024 May 20;7(5):e2411699. doi: 10.1001/jamanetworkopen.2024.11699 (PMC11107302; doi:10.1001/jamanetworkopen.2024.11699)
Supplement: Supplement 2. — Data Sharing Statement [file jamanetwopen-e2411699-s002.pdf]

Data Sharing Statement

Glazer. Maternal Characteristics and Rates of Unexpected Complications in Term Newborns by Hospital. *JAMA Netw Open*. Published online May 20, 2024. doi:10.1001/jamanetworkopen.2024.11699

Data
Data available: No

Additional Information
Explanation for why data not available: Data use agreements do not allow for sharing of individual patient data.
